# A contemporary comparison of laparoscopic versus open partial nephrectomy for renal cell carcinoma

**DOI:** 10.1186/s12894-024-01423-w

**Published:** 2024-03-12

**Authors:** Edouard Nicaise, Adam S. Feldman, Andrew Gusev, Alice Yu, Naren Nimmagadda, Matthew F. Wszolek, Francis McGovern, Michael L. Blute, Douglas M. Dahl

**Affiliations:** 1 Department of Urology, Massachusetts General Hospital, 55 Fruit Street GRB 1102, 02114 Boston, MA USA; 2 Department of Genitourinary Oncology, H. Lee Moffitt Cancer Center, Tampa, FL USA; 3 The James Buchanan Brady Urological Institute, Johns Hopkins University School of Medicine, Baltimore, MD USA

**Keywords:** Laparoscopy, Open surgery, Partial nephrectomy, Renal cell carcinoma, Renal function, Outcomes

## Abstract

**Purpose:**

To analyze surgical and oncologic outcomes of patients undergoing open partial nephrectomy (OPN) versus laparoscopic partial nephrectomy (LPN) for treatment of renal cell carcinoma (RCC).

**Methods:**

We retrospectively investigated our institutional RCC database for patients who underwent PN for RCC from 1997 to 2018. Decision for technique was at the discretion of the operating urologist, following practice patterns and training history. Outcomes analyzed included pre/peri/post-operative parameters, pathologic outcomes, and disease recurrence rates.

**Results:**

1088 patients underwent PN from 1997 to 2018. After exclusionary criteria, 631 patients who underwent 647 unique PNs for a total of 162 OPN and 485 LPN remained. Baseline, pre-op, and pathologic characteristics were not statistically different. Surgical time was lower in laparoscopic cases [185 vs. 205 min] (*p* = 0.013). Margin involvement was not statistically different; LPN had lower estimated blood loss (EBL) [150 vs. 250 mL] (*p* < 0.001) and longer ischemia time [21 vs. 19 min] (*p* = 0.005). LPN had shorter length of stay [2 vs. 4 days] (*p* < 0.001), fewer overall complications (*p* < 0.001), and no significant difference in high-grade complications [2.89 vs. 4.32%] (*p* = 0.379). Fewer LPN patients developed metastases [1.65 vs. 4.94%] (*p* = 0.0499). Local recurrence rates were not statistically different [1.24 vs. 3.09%] (*p* = 0.193). Renal function was equivalent between cohorts post-operatively.

**Conclusion:**

Long-term oncologic outcomes were not significantly different between LPN versus OPN, with no statistical difference in patient and tumor characteristics. LPN was associated with lower EBL, shorter length of stay, and lower overall complication risk. Renal function was not significantly different between cohorts.

## Introduction

Over the past 20 years the overall incidence of renal masses has notably increased in the USA [1]. Nephron-sparing surgery (NSS) has become the main approach for the treatment of small to mid-sized masses (≤ 7 cm) given the impact radical nephrectomy (RN) can have on long-term renal function [2–5]. Greater usage of contemporary abdominal imaging has helped identify these masses amenable to partial nephrectomy (PN) [6]. Evidence supports the advantage PN has over RN when reducing the risk of surgically induced chronic kidney disease (CKD) [4]. PN also has equal oncological, post-operative, and overall survival outcomes compared to RN for the treatment of small to midsize renal masses [7]. The development of both laparoscopic and robot-assisted approaches to PN has made these techniques more appealing and increasingly used in comparison to an open approach [8]. Minimally invasive techniques for PN from earlier reports demonstrated more complications and longer operative times than open approaches requiring attention to patient selection, however, newer series have now shown evidence of decreasing complication rates, shorter ischemia time and shorter hospital stays in comparison to open surgeries [9–12]. The primary barrier to widespread adoption of minimally invasive approach has been technical difficulty, but advancements in laparoscopic techniques and training have helped promote its popularity and bridge this gap.

Contemporary data comparing robot-assisted to laparoscopic approaches to PN exist, whereas reviews of laparoscopic compared to open approaches are often older and from outdated series [13, 14]. Some of these reviews still question the efficacy of laparoscopic approaches compared to open approaches for partial nephrectomy [9, 14]. We analyzed peri-operative and postoperative outcomes of pure laparoscopic vs. open techniques for partial nephrectomy. We assessed the frequency and severity of postoperative complications, length of hospital stays, impact on renal function as measured by estimated glomerular filtration rate (GFR) and the CKD Stage, and the rates of locally recurrent and metastatic disease.

## Materials and methods

**Fig. 1:**
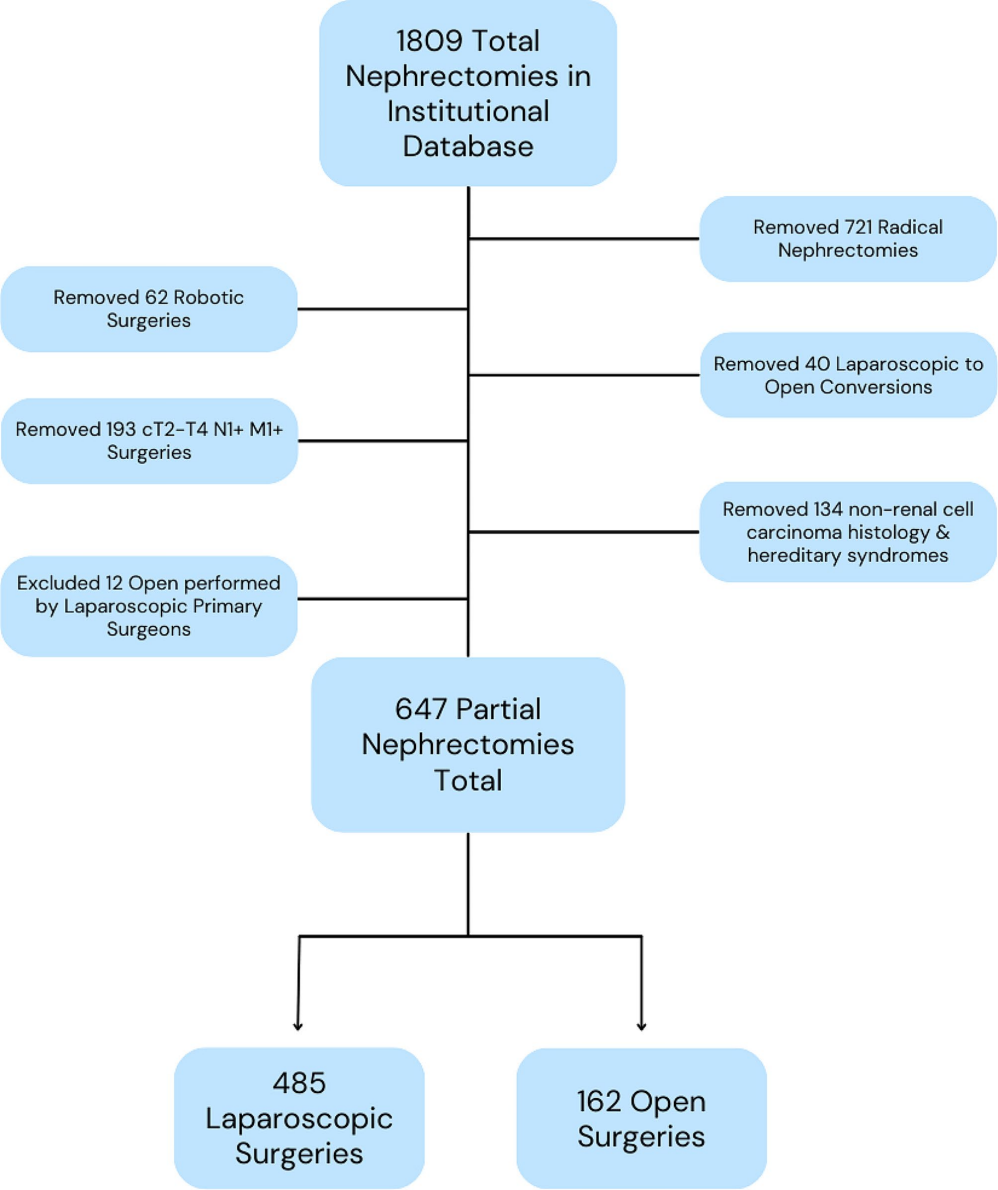
Flowsheet demonstrating inclusion and exclusion criteria for the study

The institutional review board approved this retrospective study. We investigated our institutional renal cell carcinoma (RCC) database for patients who underwent partial nephrectomy from 1997 to 2018. Only clinical stage T1 tumors were included. Exclusion criteria were patients who had undergone robot-assisted laparoscopic partial nephrectomy, benign surgical pathology, diagnosis of hereditary/genetic RCC syndrome, and multiple tumors at initial presentation. Operations converted from laparoscopic to open were excluded from the final analysis (*n* = 40). 3 urologic surgeons, with a range of 7 to 20 years in practice, primarily performed the laparoscopic cases, whereas 2 urologic surgeons, with a range of 30 to 40 years of experience performed the open cases. There were 12 open surgeries performed by primarily laparoscopic surgeons that were excluded. Ten of these patients had moderate RENAL nephrometry scores rated as majority or entirely endophytic (7–9), one had high complexity (10–12), and the remaining could not be characterized. This provided a total of 631 patients who underwent 647 PN: 485 LPN and 162 OPN. Figure 1 documents the patient exclusion and inclusion flowchart for this study.

**Fig. 2:**
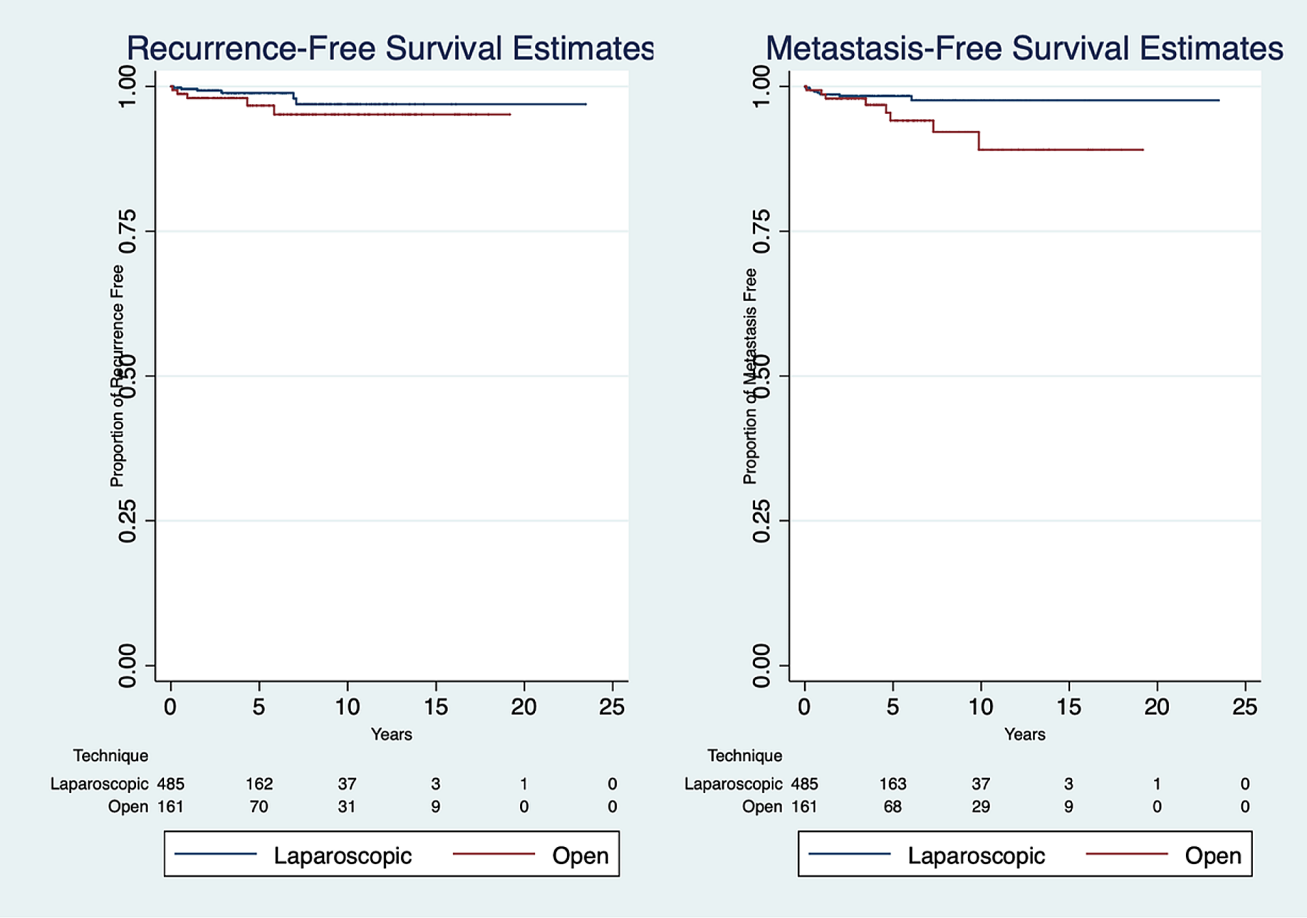
Recurrence-free **(A)** and Metastasis-Free **(B)** Survival Estimates for LPN vs. OPN

**Fig. 3:**
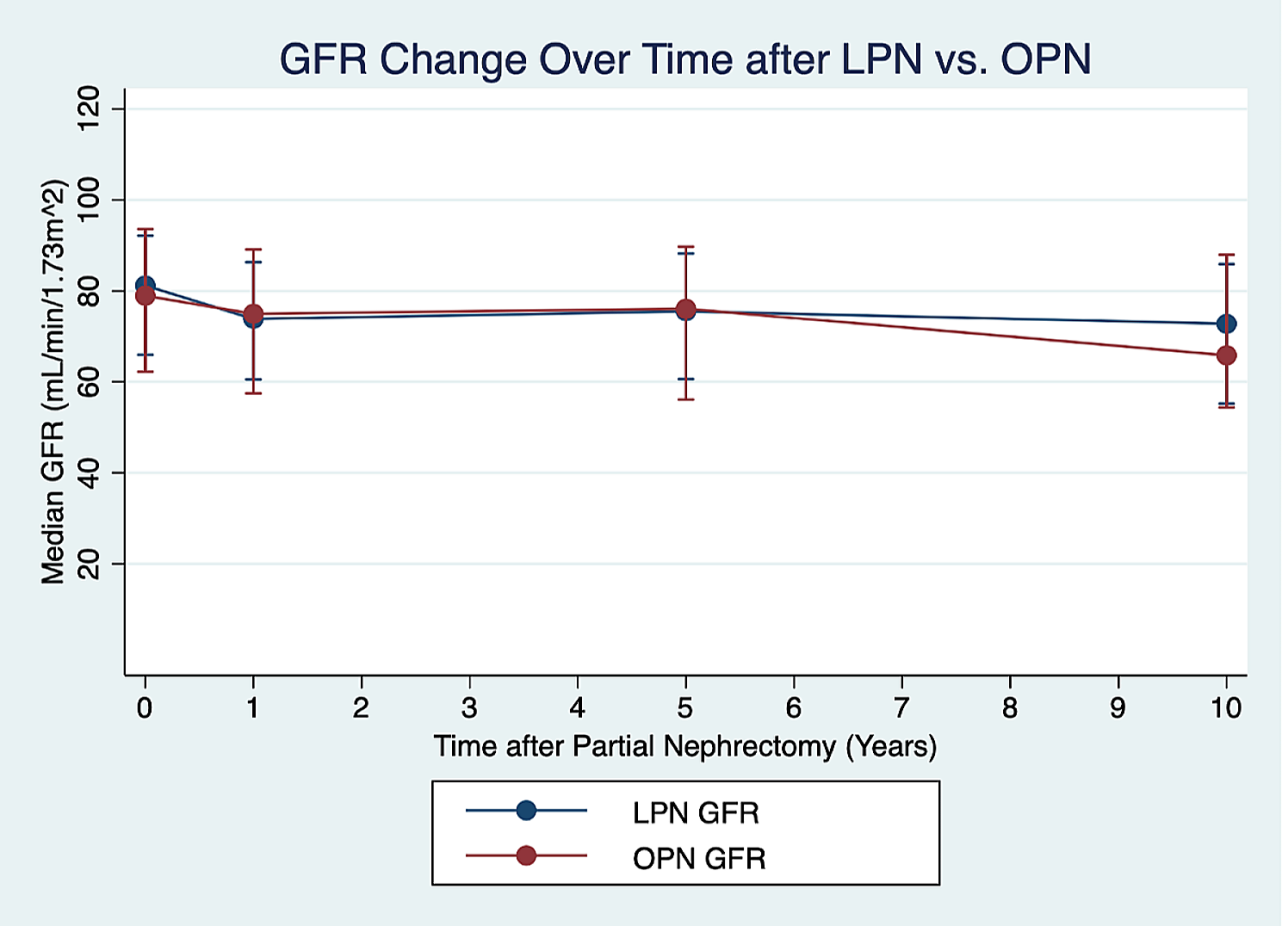
Median GFR Change over time after LPN vs. OPN

Preoperative variables included age at time of surgery, sex, BMI, tumor size according to most recent imaging prior to surgery, clinical stage, RENAL score, American Society of Anesthesiologists Physical Status (ASA Score), estimated GFR, and CKD Stage. Tumor characteristics were obtained from preoperative abdominopelvic computerized tomography and/or magnetic resonance imaging. Size was recorded as the longest single dimension of the lesion as measured by the radiologist. Clinical staging was performed according to the TNM RCC staging system. RENAL Nephrometry scoring was retrospectively determined based on review of the imaging and identified tumor features according to previously published criteria [15]. Scores were classified as Low (sum 4–6), Intermediate (sum 7–9) or High (sum 10–12) grade. The ASA Score was classified according to the documented definitions. Scores were classified as low risk (ASA Score I-II) or high risk (ASA Score III-IV). GFR was estimated using the CKD-EPI Creatinine equation with race optional. CKD Staging was based on the guidelines introduced by the National Kidney Foundation (NKF) Kidney Disease Outcomes Quality Initiative (KDOQI). Stages were then classified as low grade (CKD Stages G1-G2) and high grade (CKD Stages G3a-G5) using the cutoff GFR of 60 mL/min/1.73m2.

The decision for OPN vs. LPN was determined based on surgeon experience. Surgical characteristics included operative time, type of arterial clamping, ischemic time, estimated blood loss, presence of positive surgical margins and intra-operative complications. Disease outcomes included rates of local recurrence and rates of distant metastatic disease. Local recurrence was defined as tumor recurrence in the ipsilateral site of prior partial nephrectomy or adjacent perinephric tissue. Distant metastatic disease was evidenced by extrarenal imaging and occasionally confirmed with extrarenal biopsies. Abdominopelvic cross-sectional imaging and chest CT or XR were obtained at 6–12-month intervals following surgery.

Postoperative complications were defined as having occurred within 30 days status post PN. Complications were also analyzed with the Clavien-Dindo grade system and separated into low-grade (grade ≤ 2) vs. high-grade (grade ≥ 3a) complications.

Post-operative CKD Staging and estimated GFR were determined from serum creatinine levels measured at 3 distinct time ranges: within 1 year from surgery, within 1–5 years and then 5 + years out from surgery if available based on compliance with follow-ups and laboratory appointments.

Continuous data were compared using the Two-Sample Wilcoxon Rank Sum Test. Ordinal and categorical variables were compared with Pearson Chi-Square test or Fischer’s Exact Test, if more than 20% of cells were expected to have expected frequencies ≥ 5. Kaplan-Meier analysis with log-rank comparison was performed to compare 10-year local recurrence-free and metastasis-free survival rates. All analysis was performed using Statistical/Data Analysis Special Edition v.15.1 (StataSE, College Station, Texas, USA) with a two-sided *p* < 0.05 considered to indicate statistical significance.

## Results

There were 1088 patients who underwent PN from 1997 to 2018. Following exclusion criteria, a total of 631 patients with 647 PN cases, 162 OPN, and 485 LPN, underwent partial nephrectomy for confirmed RCC with a median follow-up of 3.4 (IQR: 1.6–6.8) years after surgery (LPN: 3.2 [1.5–6.3]; OPN: 4.0 [1.8–8.2]).

Tables 1 and 2 list the demographic, tumor, operative, perioperative, and post-operative data for the patients who underwent LPN or OPN for confirmed RCC from 1997 to 2018 at our institution. Patient baseline characteristics of age, BMI, sex, ASA PS, tumor sizes, clinical stage, and RENAL Nephrometry Score were not statistically different between the two cohorts. There was a higher percentage of entirely endophytic tumors in the OPN group (16 [10.53%] vs. 19 [4.24%], *p* = 0.017), although no difference was observed with nearness to collecting system or location relative to polar lines.

**Table 1:** Baseline Characteristics

	**Laparoscopic**			Open			***P*** value
	**N = 485**			**N = 162**			
	Data are presented as median (IQR) or n (%)						
**Age at surgery (years)**	59		(51–68)	57		(49–67)	0.183
**Sex**							
Male	346		(71.34%)	106		(65.43%)	0.156
Female	139		(28.66%)	56		(34.57%)	
**BMI (kg/m^2)**	29		(26–33)	29		(27–33)	0.166
**ASA Score**							
Low Grade (1–2)	365		(75.88%)	102		(70.83%)	0.221
High Grade (3–4)	116		(24.12%)	42		(29.17%)	
**Pre-Op GFR (mL/min/1.73 m2)**	81.30		(66.26–92.16)	78.08		(62.69–93.38)	0.560
**Pre-Op CKD Staging**							
LG (Stages 1–2) HG (Stages 3–5)	414 71		(85.36%) (14.64%)	130 31		(80.75%) (19.25%)	0.164
**Tumor Size (cm)**	2.6		(1.9–3.7)	2.8		(2.1-4.0)	0.142
**Clinical Stage**							
cT1a	398		(82.06%)	124		(76.54%)	0.123
cT1b	87		(17.94%)	38		(23.46%)	
**RENAL Nephrometry Score**							
Low (4–6)	252		(56.25%)	83		(54.61%)	0.229
Moderate (7–9)	187		(41.74%)	62		(40.79%)	
High (10–12)	9		(2.01%)	7		(4.61%)	
**Exophytic versus Endophytic**							
≥ 50% exophytic < 50% exophytic Entirely endophytic	311 118 19		(69.42%) (26.34%) (4.24%)	94 42 16		(61.84%) (27.63%) (10.53)	**0.017**
**Nearness to Collecting System (mm)**	10		(2–20)	10		(2–20)	0.304
**Location Relative to Polar Lines**							
Entirely above/below < 50% across Majority between/interpolar	196 146 106		(43.75%) (32.59%) (23.66%)	61 60 31		(40.13%) (39.47%) (20.39%)	0.304
**Pathologic Stage**							
pT1a	383	(78.97%)		123	(75.93%)		0.736
pT1b	75	(15.46%)		29	(17.90%)		
pT2a	1	(0.21%)		0	(0%)		
pT2b	0	(0%)		0	(0%)		
pT3a	26	(5.36%)		10	(6.17%)		
**Tumor Grade**							
Low Grade (1–2) High Grade (3–4) Not Reported	39 3 443	(8.04%) (0.62%) (91.34%)		19 3 140	(11.73%) (1.85%) (86.42%)		0.107
**RCC Subtype**							
Clear Cell	293	(60.41%)		99	(61.11%)		0.985
Papillary	24	(4.95%)		9	(5.56%)		
Papillary Type 1	71	(14.64%)		21	(12.96%)		
Papillary Type 2	15	(3.09%)		6	(3.70%)		
Chromophobe	44	(9.07%)		14	(8.64%)		
Other Unclassified	34 4	(7.01%) (0.82%)		11 2	(6.79%) (1.23%)		

**Table 2:** Operative and post-operative results

	**Laparoscopic**		Open		*P* value
	**N = 485**		**N = 162**		
	Data are presented as median (IQR) or n (%)				
**Surgical time (mins)**	185	(150–235)	205	(168–250)	**0.013**
**Clamping Type**					
Main artery only	353	(72.78%)	60	(37.04%)	**< 0.001**
Artery and Vein	29	(5.98%)	39	(24.07%)	
Selective arterial	73	(15.05%)	4	(2.47%)	
None	30	(6.19%)	56	(34.57%)	
Unknown	0	(0%)	3	(1.85%)	
**Ischemic Time (mins)**	21	(17–27)	19	(14–25)	**0.005**
**EBL (mL)**	150	(100–300)	250	(150–425)	**< 0.001**
**Margins**					0.704
Involved	15	(3.09%)	6	(3.70%)	
Uninvolved	470	(96.91%)	156	(96.30%)	
**Length of Hospital Stay (days)**	2	(2–3)	4	(3–5)	**< 0.001**
**Complications**					
None	414	(85.36%)	118	(72.84%)	**< 0.001**
Any	71	(14.64%)	44	(27.16%)	
**Clavien-Dindo Grade**					
HG (≥ 3) *Pneumothorax* *Hemorrhage* *Urine Leak*	14 *1* *6* 7	(2.89%)	7 *2* *3* *2*	(4.32%)	0.372
LG/None	471	(97.11%)	155	(95.68%)	
**Local Recurrence**	6	(1.24%)	5	(3.09%)	0.193
**Metastasis**	8	(1.65%)	8	(4.94%)	**0.0499**

LPN was associated with shorter operative time (*p* = 0.013), lower EBL (*p* < 0.001), fewer overall postoperative complications (14.64 vs. 27.16%, *p* < 0.001), and shorter length of stay (*p* < 0.001). Minor complications not requiring intervention included postoperative nausea and/or vomiting, ileus, urinary retention, minor renal hematoma, atelectasis, and thromboembolic problems.

OPN had less vascular clamping at the time of tumor resection (*p* < 0.001) along with shorter ischemic time if clamping was performed (*p* = 0.005). There were no significant differences in RCC Subtype (*p* = 0.985) or pathological staging (*p* = 0.736). There was no difference in the number of Clavien-Dindo Classified ≥ 3a complications between LPN and OPN (2.89 vs. 4.32%, *p* = 0.498). Primary urological complications noted were acute renal failure, urinary leakage, ureteral obstruction, hemorrhage, and urinary tract infection. Non-urological complications represented cardiac, hematological, gastrointestinal, pulmonary, and thromboembolic problems. Table 2 summarizes the types of high-grade postoperative complications encountered. The three primarily reported complications requiring re-intervention were pneumothorax, urine leak, and hemorrhage. No difference was observed in regard to positive margins on final pathology: 15 LPN patients compared to 6 OPN patients (3.09 vs. 3.70%, *p* = 0.704). Local recurrence occurred in 6 LPN (1.24%) and 5 OPN (3.09%) cases, with no statistical significance (*p* = 0.193). Figure 2 demonstrates no difference in 10-year recurrence-free survival rate of LPN compared to OPN. However, there was a significant difference in the 10-year rate of metastasis between LPN vs. OPN (1.65 vs. 4.94%, *p* = 0.0499).

Table 3 summarizes the renal function of both cohorts pre and post-partial nephrectomy. Pre-operative GFR and CKD staging were not significantly different between LPN vs. OPN. There were no differences in postoperative GFR between LPN vs. OPN within the 1-year mark (73.94 vs. 75.01 mL/min/1.73m2, *p* = 0.815), between 1 and 5 years post-operatively (75.81 vs. 77.85, *p* = 0.822), or after 5 + years post-operatively (73.11 vs. 67.64, *p* = 0.406) (Fig. 3). There was also no difference in post-operative renal function according to CKD staging. During the period of follow-up, 3 patients (2 LPN, 1 OPN) progressed to stage 5 CKD or end-stage renal disease requiring dialysis.

**Table 3:** Post-operative renal function results

	**Laparoscopic**		Open			*P* value	
	**N = 485**		**N = 162**				
	Data are presented as median (IQR) or n (%)						
**Post-Op GFR ≤ 365 Days (mL/min/1.73 m2)**	73.94	(60.63–86.38)	75.01	(58.82–89.46)	0.815		
**Post-Op CKD Staging ≤ 1 year**							
LG (Stages 1–2) HG (Stages 3–5)	367 117	(75.83%) (24.17%)	117 45	(72.22%) (27.78%)	0.360		
**≤ 1 year % Change in GFR from baseline**	-7.58%	(-17.35 - +1.33%)	-4.34%	(-14.19 - +4.91%)	0.112		
**Post-Op GFR 1–5 years (mL/min/1.73 m2)**	75.81	(60.80-88.25)	77.85	(56.57–89.71)	0.822		
**Post-Op CKD Staging 1–5 years**							
LG (Stages 1–2) HG (Stages 3–5)	221 68	(76.47%) (23.53%)	61 27	(69.32%) (30.68%)	0.176		
**1–5 years % Change in GFR from baseline**	-7.19%	(-17.39 - +2.36%)	-7.82%	(-17.32 - -0.62%)	0.686		
**Post-Op GFR > 5 years (mL/min/1.73 m2)**	73.11	(55.54–85.99)	67.64	(54.27–88.48)	0.406		
**Post-Op CKD Staging > 5 years**							
LG (Stages 1–2) HG (Stages 3–5)	103 44	(70.07%) (29.93%)	42 24	(63.64%) (36.36%)	0.352		
**> 5 years % Change in GFR from baseline**	-6.75%	(-18.30 - +4.89%)	-10.46%	(-19.49 - -0.94%)	0.113		
**Progression to HG CKD**	134	(27.69%)	57	(35.19%)	0.267		
**Progression to Stage 5 CKD**	2	(0.41%)	1	(0.62%)	0.383		

## Discussion

The incidence of small renal masses has increased in the USA over the past 20 years [1]. Partial nephrectomy has proven to be an effective and equivalent oncologic treatment method in comparison to radical nephrectomy, without significant long-term impact on renal function [3]. Minimally invasive alternatives to OPN, including laparoscopic and robot-assisted PN, have become accepted. Recent studies have demonstrated improvements in overall complication rates, ischemic time, EBL, and length of stay, but there had been concerns regarding positive margin rates, local recurrence, and metastasis rates [16–19]. Contemporary studies have compared these long-term oncological outcomes between robot-assisted and laparoscopic PN, with favorable results for both minimally invasive techniques. However, there are fewer studies featuring comparisons of laparoscopic to open technique.

Our data demonstrate that LPN has improved perioperative outcomes as measured by the shorter operative time, lower EBL, decreased postoperative complication rate, and shorter length of stay. This could be explained by the size and complexity of the tumors selected for open resection, but our data showed no statistically significant difference in tumor size, clinical staging, or RENAL Nephrometry Score between the two cohorts. However, there was a statistically higher percentage of entirely endophytic tumors in the OPN cohort, which may have contributed to the difference in perioperative outcomes. Although remaining tumor-related factors, including nearness to collecting system and polar line location were not significantly different. The ASA Score was not different between both cohorts as well. Although LPN is associated with lower overall postoperative complications, there was no difference in the rate of higher-grade complications with the most common being hemorrhage and urine leak.

Prior studies have often demonstrated LPN is associated with longer warm ischemic time [10, 12, 20], which was also identified in our cohort (21 vs. 19 min). Although this was statistically significant, this difference is still relatively small and may not, in effect, be clinically significant. Options to further reduce warm ischemic time include preoperative superselective arterial embolization [21]. This technique can allow for LPN to be performed off clamp with minimal blood loss (median 106 mL) and a low percent decline in GFR (5%) at 1 year [22]. There was no difference in GFR between our open and laparoscopic cohorts following PN. The overall percent decline in GFR was 7–11% compared to the initial preoperative value. Only 3 patients total [2 LPN (0.4%) vs. 1 OPN (0.6%)] progressed to end-stage renal disease (ESRD) during follow-up, defined as CKD Stage 5, or need for dialysis.

There were early concerns for higher risk of local recurrence and distant metastasis rates in LPN vs. OPN, however, more recent studies have demonstrated no statistical differences in these oncologic measures [9–14, 20, 23, 24]. Our data also supports the noninferiority of LPN with no difference in positive surgical margin, local recurrence, or metastasis rates. OPN displayed higher rates of distant metastasis despite no statistical difference in tumor sizes, clinical staging, and RENAL Scores. Although there was a slight difference in tumor size (+ 0.2 cm larger among OPN), this is unlikely to be clinically significant enough to explain the higher rate of metastasis. However, there was a higher rate of entirely endophytic tumors in the OPN cohort, which could explain the greater aggressiveness. Central location and endophytic growth has shown to be associated with higher nuclear grading which may reflect this increased risk of metastasis [25].

Limitations to this study included the retrospective nature of the analysis and a wide range in follow-up time for both cohorts. Tumor grading was missing from the majority of final surgical pathology reports, which could impact survival outcomes. This study will strengthen the current research on the efficacy of LPN in treating renal masses with improved perioperative and equivalent oncological outcomes. It can help demonstrate the noninferiority of LPN, particularly at sites where robot-assisted PN is unavailable given limitations in access or training.

## Conclusions

Our results show that pure LPN has non-inferior oncologic outcomes to OPN. Laparoscopic technique was associated with shorter operative time, lower EBL, shorter length of stay, and lower overall complication risk. Small, insignificant differences in tumor size, ischemia time, and rate of metastasis are of unlikely clinical significance. Renal function was equally maintained in both cohorts.

## Data Availability

Data regarding any of the subjects in the study has not been previously published unless specified. Data will be made available to the editors of the journal for review or query upon request. Raw data and statistical code can be provided upon request.
